# Implementation of shared decision making in rheumatoid arthritis: study protocol for RAiSeD (Rheumatoid Arthritis Shared Decision Making) stepped wedge, cluster-randomized trial

**DOI:** 10.1186/s13063-025-09015-1

**Published:** 2025-09-29

**Authors:** Jennifer L. Barton, Meike Niederhausen, Anaïs Tuepker, Gabriela Schmajuk, Joshua Baker, Travis I. Lovejoy, Benjamin J. Morasco, Marleen Kunneman, Isabelle Scholl

**Affiliations:** 1 VA Portland Health Care System, Portland, USA; 2 Oregon Health & Science University, Portland, USA; 3 San Francisco VA Health Care System, San Francisco, USA; 4 Corporal Michael J. Crescenz VA Medical Center, Philadelphia, USA; 5 Leiden University Medical Center, Leiden, Netherlands; 6 Mayo Clinic, Rochester, USA; 7 University Medical Center Hamburg-Eppendorf, Hamburg, Germany

**Keywords:** Rheumatoid arthritis, Shared decision making, Decision aid, Clinician training, Quality of care, Veterans

## Abstract

**Background:**

Rheumatoid arthritis (RA) impacts quality of life causing disability and increased mortality. Treatment decisions are complex and require individualization. Shared decision making (SDM) is the first principle of RA treat-to-target guidelines, but uptake is suboptimal. We aim to evaluate the effectiveness of a multicomponent SDM intervention on RA disease activity and explore the early implementation of the intervention within three geographically diverse rheumatology services.

**Methods:**

The RAiSeD trial uses a stepped-wedge, cluster-randomized trial design at three U.S. Veterans Health Administration rheumatology clinics, targeted to enroll more than 400 patients and over 45 clinicians. The multicomponent SDM intervention consists of three parts: (1) rheumatology clinician training and a pocket card on SDM and fostering choice awareness (“acknowledging when there is more than one sensible option available to address a patient’s situation”), (2) RA patient activation using the AskShareKnow questions, and (3) a point-of-care decision aid (RA Choice) and medication summary guide. We will conduct a mixed-methods outcomes and process evaluation. Outcomes will be evaluated during a pre-intervention (usual care) and intervention period. The primary outcome is disease activity as measured by the validated Clinical Disease Activity Index (CDAI), with secondary outcomes of RA knowledge and medication adherence. SDM will be measured by two brief, validated patient-reported measures. A subgroup of clinic visits will be audio-recorded and clinicians’ efforts to involve patients in SDM will be assessed. The implementation process will be evaluated using stakeholder interviews and field notes at each of the three sites.

**Discussion:**

This study is the first multi-site trial of a multicomponent intervention to facilitate SDM among veterans with RA. We expect to improve uptake of SDM across geographically distinct rheumatology clinics and hypothesize that patients exposed to the interventions will have a greater decrease in disease activity and an increase in knowledge of RA medications compared to usual care. Insights gained from this study will inform broader dissemination and implementation of SDM across VA rheumatology clinics and beyond, with the goal of improving quality of care for all persons with RA.

**Trial registration:**

ClinicalTrials.gov NCT05530694. Registered on September 7, 2022.

**Supplementary Information:**

The online version contains supplementary material available at 10.1186/s13063-025-09015-1.

## Background

Rheumatoid arthritis (RA) is a systemic, chronic, autoimmune disease which affects up to 1% of the population, causes significant disability, and accounts for up to $20 billion in societal costs annually [1]. Over the past two decades, advancements in the treatment of RA with biologic disease-modifying anti-rheumatic drugs (DMARDs), targeted synthetic DMARDs, and combination therapy brought the goal of clinical remission within reach. Despite this progress, disparities in outcomes and access to therapy persist among traditionally disadvantaged groups with RA and those with limited health literacy [2–6]. Non-adherence to DMARDs may play a role in observed disparities. Reports of RA medication treatment among ethnically diverse and low-income patients found as little as 21% had adequate adherence [7]. Lower adherence appears to be related, in part, to undisclosed and unconsidered patient preferences and values as well as perceived treatment inefficacy [8, 9].

Shared decision making (SDM) is a process whereby both patient and clinician take into account the best available evidence of risks and benefits across available options, as well as patient values and preferences when making medical decisions [10, 11]. Effective methods to foster SDM include decision aids and clinician training [12]. A 2024 systematic review of 209 studies of more than 100,000 participants showed that decision aids improve patient knowledge, enhance communication, and involve patients in decisions, but highlighted gaps around efficacy for patients with limited health literacy [13].

The cornerstone of RA treatment is methotrexate, and up to one-third of patients will achieve remission or low disease activity with methotrexate monotherapy. The other two-thirds—up to 1 million United States (U.S.) adults with RA—will face decisions to modify therapy. Identifying the right therapy for the right patient from an ever-growing list of potentially toxic yet effective therapies requires individualized conversations between patients and clinicians.

SDM is prioritized by RA treatment guidelines in the U.S. and Europe and is the first overarching principle of treat to target guidelines [14]. Despite its importance, few reliable SDM tools or trainings exist for rheumatologists and persons with RA, especially those with limited health literacy. In a prior study, one-third of U.S. adults with RA reported suboptimal communication around SDM (response of never/rarely/sometimes to two questions: “How often did you and your doctors work out a treatment plan together?” and, “If there were treatment choices, how often did doctors ask if you would like to help decide your treatment?”), particularly among those with limited health literacy and low trust in physicians [15]. A 2019 study of audio-recorded, routine RA visits found the level of SDM to be very low, reinforcing a need for training and tools to improve this gap [16]. A pilot study aimed at testing the effectiveness of a low literacy, multilingual decision aid for RA (RA Choice) demonstrated improved knowledge and reduced decisional conflict among persons with limited health literacy, limited English language proficiency, and those from non-white/non-Hispanic racial and ethnic groups [17]. Interventions which combine clinician training, patient activation, and point-of-care decision aids show greater impact on SDM outcomes [18–20].

Of note, no SDM interventions have been tested among veterans with RA. Veterans with RA, who are predominantly male and have a two-fold higher risk of death over a 10-year period compared with the general non-RA population [21], represent a unique population in which to study the effectiveness of an intervention designed to improve communication and patient engagement. Characteristics of veterans who utilize VA for care (older, male, higher rates of tobacco use, more comorbidities) have been associated with poorer function and poorer quality of care in the general RA population (e.g., less likely to receive DMARD; [22, 23]). Multiple studies from a variety of populations indicate that RA prevalence, severity, and disease-related mortality disproportionately impact individuals of lower socioeconomic status [24]. It remains unknown the impact of an SDM intervention on disease outcomes and adherence among veterans with RA.

Therefore, we aim to evaluate the effectiveness of a multi-component SDM intervention (clinician training, patient activation, RA Choice decision aid) in a stepped-wedge, cluster-randomized controlled trial on outcomes of disease activity, RA knowledge, and adherence. We hypothesize that during SDM intervention phases, veterans will have lower disease activity compared to during usual care periods and will be more likely to experience clinically important differences in a standard disease activity index. We will also evaluate the effectiveness of a multi-component intervention to facilitate SDM using both patient-reported measures and a measure of direct observation of audiotaped encounters and conduct a qualitative evaluation of the SDM intervention and local implementation to inform future dissemination. We hypothesize greater uptake of SDM in enrolled clinics during the intervention phase relative to the usual care phase. In this manuscript, we describe the protocol of the RAiSeD trial for veterans with RA.

## Methods

### Study design

**Fig. 1:**
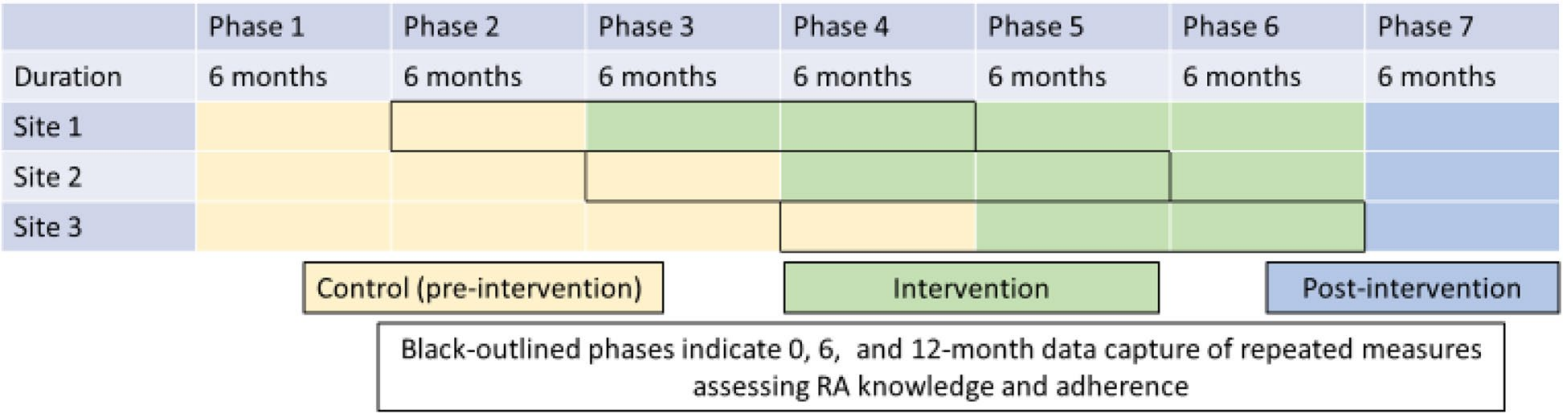
Study design: stepped wedge, randomized controlled trial of multicomponent SDM intervention for persons with RA

This study will evaluate the effectiveness of a multicomponent intervention to foster SDM through an open cohort, stepped-wedge cluster-randomized trial. The stepped-wedge design is a form of a cluster, randomized study where the intervention is launched in each cluster over several set time periods. The order of the start time of the intervention for each cluster is determined at random. In this design, there is an initial period in which no clusters (clinical sites) receive the intervention (pre-intervention or usual care) and by study end, all clusters have received the intervention. The stepped-wedge design is particularly suitable to evaluate interventions during routine care [25]. Specifically, the advantages of the stepped-wedge cluster design include studying a phased roll-out (as proposed) and maximizing statistical power due to within cluster comparisons with sites acting as their own control, in addition to between cluster comparisons [26]. The design also allows each site to act as its own control. The phased roll-out allows for improvements in the intervention or its delivery where needed prior to the next study phase [Fig. 1]. For preparing this protocol report, we followed the CONsolidated Standards of Reporting Trials (CONSORT) extension for stepped wedge cluster randomised trials where applicable (see Additional file 1; [27]).

### Study setting

The study will take place at three VA rheumatology clinics located in the United States: Portland, OR; San Francisco, CA; Philadelphia, PA.

### Eligibility criteria

Rheumatology clinicians (attendings, fellows or advanced practice partners—nurse practitioners or physician assistants) at the respective clinics are eligible for participation. Non-clinician participants who have held a leadership position within their respective institution or who have worked in the rheumatology clinic setting for a minimum of 12 months prior to enrollment are eligible for qualitative interviews to evaluate implementation.

Patients are eligible if they are 18 years of age or older, have RA (as defined using an administrative data definition of RA—at least 2 visits with RA ICD10 codes including their last rheumatology clinic visit and the use of a DMARD for at least 180 days), receive rheumatologic outpatient care at participating clinics and were seen at least once in the preceding 12 months, are English-speaking, and have moderate to high disease activity within 18 months prior to enrollment. The routine measurement of disease activity for persons with RA is part of usual care. All three study sites routinely measure disease activity using one of several American College of Rheumatology (ACR)-endorsed composite measures (e.g., Clinical Disease Activity Index or CDAI, 28 joint Disease Activity Score or DAS28, Routine Assessment of Patient Index Data 3 or RAPID3; [28].

Target sample size of clinicians (*n* = 33) and eligible RA patients (*n* = 375) was based on personal communication with local site clinician leaders and review of electronic health records over a twelve-month period. Total clinicians across the three sites was 33. Established patients routinely have at least one clinic visit every 6 months, which is the duration of each study phase. Thus, with an anticipated 375 patients with moderate to high disease activity, we expect about 2250 patient visits over the 6 phases, which is on average 11 patients per clinician per phase.

### Procedures and participant identification and recruitment

#### Procedures

Eligible clinicians or clinician leadership are invited to participate in one or more aspects of the study: (1) audio recording of clinic visit; (2) two semi-structured interviews (once in the usual care period, once in the post-intervention period).

**Fig. 2:**
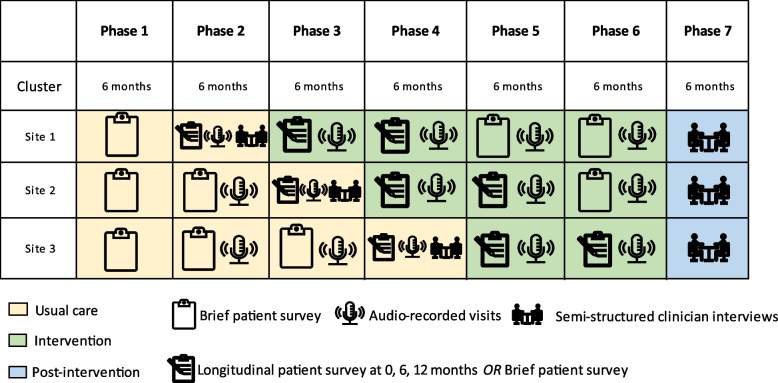
RAiSeD study phases and data collection

**Fig. 3:**
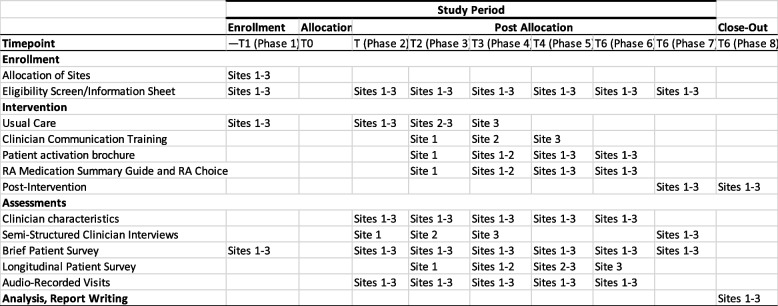
SPIRIT figure

Eligible patient participants are invited to participate in one or more aspect of the study (Fig. 2 and SPIRIT Fig. 3): (1) complete a brief survey after a clinic visit, (2) complete a longer survey at three time points (see below), (3) audio recording of clinic visit.

A convenience sample of patients recruited from each site during the site’s last 6-month pre-intervention phase are invited to complete the brief survey plus measures of RA medication knowledge and adherence (referred to as the “longer survey”) at three measurement time-points: baseline (usual care), 6-months (intervention), and 12-months (intervention).

To assess clinicians’ behavior to involve patients in SDM from an observer’s view, rheumatology visits are audio recorded for a sub-sample of patient participants. A convenience sample of up to 5 patients per clinician during each of the pre-intervention and intervention study periods is recruited.

#### Participant identification and recruitment

Eligible clinicians will be recruited through email invitation by site study coordinators to participate in the study. They will be provided an IRB-approved information sheet and relevant study description as well as contact information. In any instances of audio recording clinic visits, study team staff will record participants’ (both clinician and patient) consent to be recorded.

Eligible patient participants will be identified using the VA electronic health records database, Corporate Data Warehouse (CDW). Using an administrative definition of RA, analysts will identify a cohort of patients with upcoming clinic appointments. Information on disease activity will be collected through the CDW, a review of charts in the electronic health record by study staff, or from disease activity assessments collected at the time of the visit as part of usual care. Once patients are identified as eligible, research staff will mail a letter to introduce the study and provide contact information for study team members, or staff will approach patients on the day of their appointment. To help ensure completion of longitudinal assessments and prevent loss to follow-up, patients will receive a $20 remuneration per survey time period (total of $60 for all completed time points).

This study was approved by the VA Central IRB (E21-09).

### Randomization

Clinic sites are randomized to the phases, using a random sequence, generated a priori by the trial biostatistician using the statistical software R [29] to determine the order in which clinical sites launch the intervention phase. The randomization schedule is implemented at the time of the clinician training at each site. For example, the clinic site enters the intervention phase on the date of the clinician training in the order in which it was randomized. There is a six-month interval between each intervention launch (step).

### Intervention

The multicomponent intervention to facilitate SDM is composed of 3 elements, none of which is currently standard of care:

1. Clinician training in fostering choice awareness (FCA). Our study team has created a generic (i.e., not rheumatology-specific), skills- and attitude-based approach for clinicians to acknowledge in their decision-making conversations with patients that (1) there is more than one option, and (2) patients’ views matter in deciding what to do next. This approach can be used during patient encounters when needed and is termed fostering choice awareness (FCA) [30, 31] The one-hour clinician training in developing FCA skills consists of 3 parts: (1) a knowledge session on evidence and guidelines on effective conversations and SDM, (2) review of behaviors using 4 separate written clinical scenarios which foster or hinder choice awareness combined with clinical simulations in which clinicians practice fostering choice awareness behaviors, and (3) a reminder card for use in future clinic visits. Written scenarios describe patient-clinician conversations around treatment decision making to prompt and probe clinicians’ experiences, improve understanding, and improve practice.
2. Patient activation. A patient-facing strategy to involve patients in care can improve patient-clinician communication [32] The strategy consists of three questions (Ask Share Know) patients can ask their clinician to help them become more involved: “1. What are my options?; 2. What are the possible benefits and harms of those options?; 3. How likely are each of those benefits and harms to happen to me?” These questions have been used in studies to promote improved communication in a number of conditions including cardiovascular disease, cancer, and women’s health [33, 34]. Dissemination of materials will be in the form of a trifold brochure handed to patients in the clinic or mailed. The original Ask Share Know pamphlet was adapted for rheumatoid arthritis [35].
3. Patient medication summary guide and point of care decision aid. RA Medication Summary Guide and RA Choice (Additional files 2 and 3). RA Choice is a within-encounter conversation aid which presents information on FDA-approved RA treatments in a set of cards. These can be used alone or in combination based on a patient’s preference, values, and the clinician’s experience. The RA medication summary guide describes options for medication, physical therapy, occupational therapy, and ways to stay healthy with diet and exercise. Tool development followed key principles of creating low literacy materials [36] that feature icons, short phrases written in plain language, and include topics of interest to RA patients faced with a medication decision. For the RAiSeD study, we include a paper-based version of the tool to be used during the clinic visit and a one-page, double-sided copy of the cards for patients to take home, or a web-based version available for virtual care visits. The tools have been updated to include new FDA-approved therapies (e.g., Janus kinase inhibitors).

#### Intervention team and training

When a site enters the intervention phase, all rheumatology clinicians at that site receive a one-hour training in fostering choice awareness led by study team members as part of each site’s usual rheumatology didactic program. Trainings will be conducted virtually at all sites. Clinicians at each site will be instructed on the use of the decision aid by a study team member. Training on RA Choice is brief (< 5 min) and clinicians will receive a copy of the RA Choice storyboard, which demonstrates its use. As reflected in a brief clinician survey (*n* = 15) at the three clinical sites, willingness to use the decision aid was high (94% answered yes to willingness to use).

#### Relevant concomitant care and interventions that are permitted or prohibited during the trial

The use of specific medications or treatments is not being restricted during this trial.

### Data collection and management

Data are collected from all sites starting during the usual care phase prior to the intervention. Data will be derived from four sources: patient surveys, CDW/medical record review, audio recordings of clinic visits, and semi-structured interviews. Patient participant data collected in the form of paper surveys at the time of clinic visits or over the phone will be entered into an electronic database by trained research staff. Paper surveys will be kept in a locked cabinet in a locked office in a secure building. Interviews will be audio recorded using VA-approved audio recording technology and transcribed verbatim using the VA Centralized Transcription Service or Microsoft Teams. After interviews with participants, audio recordings will be uploaded directly onto a network folder on a password-secure server behind the facility firewall. See Table 1 for a description of outcomes and data sources.

**Table 1:**
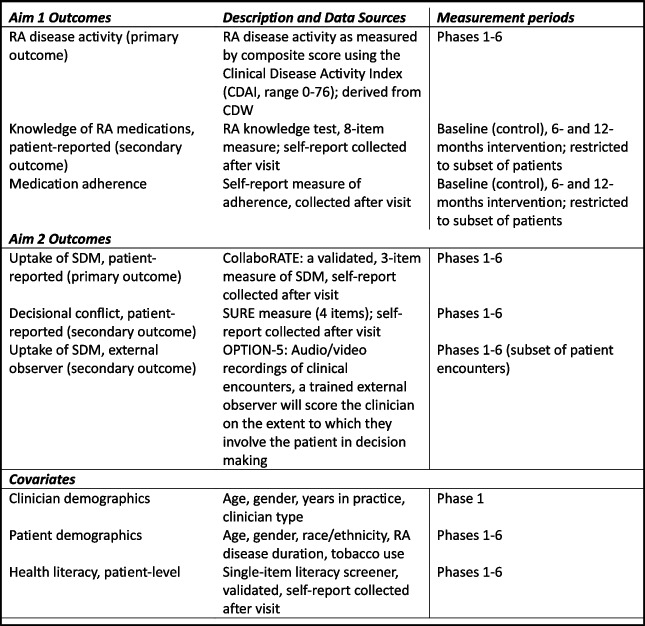
Outcome measures for Aims 1 and 2

### Measures and outcomes

#### Participant characteristics

For clinician participants who agree to participate in any aspect of the study, they are invited to complete a brief paper or REDCap survey to collect data on their age, gender, years in practice, and clinician type (MD/DO, advanced practice partner). Both the short and longer patient surveys outlined above include questions on demographic information (age, gender, race and ethnicity) and health literacy level (single-item literacy screener, [37].

*Aim 1. Evaluate the effectiveness of a multi-component SDM intervention (clinician training, patient activation, RA Choice decision aid) in a stepped-wedge, cluster-randomized controlled trial on improvement in disease activity, RA knowledge, and adherence. Primary outcome* is disease activity using an ACR-approved composite measure, the Clinical Disease Activity Index (CDAI). All sites currently collect disease activity in a systematic fashion as part of usual care. There are four components of the CDAI (patient global assessment of disease activity, physician global assessment of disease activity, 28 tender joint count, 28 swollen joint count). At each clinic visit, patients report on their global assessment of disease activity (0–10 scale) upon arrival to clinic (clinic staff hands patient a paper form to complete). The clinician performs a tender joint count (0–28) and a swollen joint count (0–28) during the visit and records these along with the physician global assessment of disease activity (0–10) (Table 1). CDAI components are summed with a total score range of 0–76 and entered into the EHR by the clinician. Cut points for disease activity categories are remission ≤ 2.8; low > 2.8–10.0; moderate > 10.0–22.0; high > 22.0. The minimal clinically important difference for a person with a moderate CDAI is 6 [38].

### Patient-reported outcomes

#### Secondary outcomes

Patient-reported knowledge of RA medications and adherence will be assessed for a subset of patients. Patients’ general knowledge about RA medications will be measured through a series of eight questions assessing basic understanding of RA and RA treatment [17]. Because the questions were designed to capture rudimentary knowledge (Additional file 4), scores of at least 7 out of 8 correct answers will be considered adequate RA knowledge. Patient-reported RA medication adherence will be assessed using a validated single-item measure: “How many times do you think you may have missed taking your pills in the last week?” [17, 39, 40]. A response of 1 or greater is considered nonadherent. Among participants taking a biologic therapy, where dosing intervals can be weekly or monthly, adherence will be measured by asking, “How many times do you think you have missed your RA injection or infusion in the past month?” Adherence will be dichotomized into either poor or adequate adherence, with poor adherence defined as missing ≥ 1 RA pills over the past week or ≥ 1 RA injections in the past month [8]. RA knowledge and adherence will be captured after the clinic visit by research staff through paper survey or by telephone.

*Aim 2: Evaluate the effectiveness of a multi-component intervention to facilitate SDM. *Outcomes for this aim will be the uptake of SDM as measured by the validated, brief, patient-reported measure CollaboRATE. The CollaboRATE scale is a 3-item measure of SDM with response options 0–9, with 0 = “no effort was made” to 9 = “every effort was made” by your clinician to help understand health issues (rheumatoid arthritis), listen to things that matter most to you about your health issues, and include you in what matters most in choosing what to do next. The CollaboRATE demonstrates discriminative validity, concurrent validity, intra-rater reliability, and sensitivity to change [41]. Due to potential ceiling effects of patient-reported experience measures, scoring involves conducting a “top score” analysis which has been shown to enhance variation in scores. The CollaboRATE outcome measure is dichotomized at whether the patient gave the top score of 9 on all three questions. The CollaboRATE will be administered to all enrolled patients cared for by enrolled clinicians who have scheduled rheumatology visits during both usual care and intervention study phases. In addition to CollaboRATE, and to have a robust examination of SDM, we will also collect a measure of patient-reported comfort with the decision about a medication change using the SURE measure. SURE is a 4-item version of the Decisional Conflict Scale and measures decisional conflict in clinical practice, reported on a 0 to 4 scale [42]. A score less than four indicates decisional conflict. The SURE measure has shown to be valid and reliable for use with adults with musculoskeletal conditions (knee/hip osteoarthritis or back pain) [43] and to be associated with less decisional regret and higher satisfaction with one’s choice of intervention [44]. The survey will be administered by research staff immediately after the clinic visit.

*Additional outcomes* will be clinicians’ behaviors to involve patients in SDM from an observer’s view, as assessed by the OPTION-5 scale using audio-recorded clinical encounters. Research staff at each site will consent eligible clinicians and patient participants who agree to the audio recording of visits. Research staff will then start the recording device in the clinic room, exit, and retrieve the device post-visit. OPTION-5 evaluates the following clinician behaviors:(1) provides explicit explanation that decisions exist that need attention and deliberation, (2) provides reassurance to the patient that the clinician will support deliberation, (3) provides information about options, (4) elicits patient’s views, preferences, priorities, and (5) integrates patient preferences into the next stage of decision making [45]. Scores for each item range from zero (no effort) to four (exemplary effort on the part of the clinician). The total score ranges from 0 to 20. To address clinician concerns of time as a barrier to SDM, we will examine time as a “balancing measure” to assess whether the intervention does or does not increase visit time using time stamps from the audio recordings during the usual care and intervention phases.

### Qualitative data

Aim 3. *Conduct a qualitative evaluation of the SDM intervention and local implementation to inform future dissemination.* We will use the Consolidated Framework for Implementation Research (CFIR) to guide an exploratory evaluation of which implementation practices work where and why, across the clinical sites. We will use CFIR as a guide for analysis, interpretation, and reporting of findings [46]. CFIR suggests that multiple factors influence the adoption and maintenance of an intervention across 5 main domains: (1) intervention characteristics (source, evidence strength/quality, adaptability); (2) outer setting (events occurring outside a clinic that influence change); (3) inner setting (characteristics of a clinic, culture, implementation climate); (4) characteristics of individuals (knowledge and beliefs, self-efficacy of staff implementing an intervention); and (5) implementation process (planning and evaluation activities). Following the CFIR framework, we will conduct assessments during the usual care phase at all 3 sites. The evaluation will enhance the likelihood of success of future implementation strategies through an analysis of the actual degree of less-than-best practice, determinants of current practice, and potential barriers and facilitators to practice change. We will also ascertain potential implementation feasibility, including perceived utility of the project.

Clinicians, rheumatology clinic staff, and system leaders will be interviewed during the usual care and post-intervention phases. Interviews will be audio recorded and transcribed verbatim. Interviews will be semi-structured using a predeveloped interview guide with questions designed to elicit data on topics such as capacity for practice change and anticipated or observed barriers and facilitators to implementation of the SDM tool. As interviews proceed, emerging themes may be incorporated into the interview guide for subsequent interviews. This process will be repeated in the post-intervention phase for each site. Study team members with qualitative training will generate field notes through direct observation at clinics during roll-out phases and while observing interactions of rheumatology clinicians with clinic staff and during data collection. Data in field notes will include the following: observations of clinic workflow and patient-staff interactions during arrival and check-in, provider discussions of patient care, and other miscellaneous observations of the clinic context that may arise, as well as memos of questions to pursue in interviews or further research. Field notes will assist with appropriate contextualization of clinic experiences, reconstruction of process timelines, and accuracy and context-rich descriptions in manuscript writing.

### Analysis plan

Data analysis. *Aim 1*. We will first summarize clinician and participant characteristics, overall and by exposure status, to evaluate potential selection bias and lack of balance and also identify covariates to include in regression models. Outcomes measured throughout the study (CDAI, CollaboRATE, SURE, and OPTION-5) will be summarized using means or proportions and respective 95% confidence intervals, stratified by the pre- and during intervention time periods. RA knowledge and medication adherence will be similarly summarized at the baseline, 6-month, and 12-month time points. We will conduct statistical analyses after completion of the intervention phase and follow intention-to-treat analyses at the clinic level. In a stepped-wedge study, distributions of results across unexposed observation periods are compared with those across exposed observation periods [26]. Primary analysis of Aim 1 will test whether average RA disease activity (CDAI) decreased after the multicomponent intervention launch, using a patient-level generalized linear mixed model (GLMM) with a continuous outcome [47]. The primary independent variable of interest is an indicator for whether an observation occurred pre- or during intervention. The coefficient and 95% confidence interval of this fixed effect will be the estimated average difference in patients’ CDAI values in the intervention versus pre-intervention phases. Other fixed effects will include time as a continuous variable, a term for time since intervention start to estimate additional changes due to intervention over time, and site. Clinician and patient will be treated as random effects since repeated measures are likely to be more correlated than information collected from different clinicians or patients. We do not expect sites or RA patients included in each phase to differ according to demographics or clinical factors, but if they do, we will add covariates to the GLMMs to adjust for these differences. We will re-evaluate the intra-cluster correlation (ICC) coefficient for reporting purposes. Secondary outcomes RA knowledge and medication adherence will be measured as binary variables at the patient level in a subset of patients followed over time and assessed at three time points (baseline usual care, and 6- and 12-month points during intervention). We use GLMMs with a logistic link function and the primary independent variable of interest will be indicators for the 6- and 12-month time points with baseline as the reference level. The coefficient and 95% confidence interval of these fixed effects will be the odds ratios of the odds of having adequate RA knowledge and adherence, respectively, at 6 or 12 months compared to baseline. The 12-month outcome is of greater interest to assess durability of intervention impact. Other fixed effects will be site and covariates as needed. Clinicians and patients will be included as random effects.

We anticipate little to no missing data regarding the primary outcome since these data are uniformly collected at each clinical site as part of standard of care for persons with RA. Patterns of missingness between variables, within sites, and within individuals will be visualized using the R package naniar. For variables with 5%–40% missingness and that are reasonably missing at random, we will use multiple imputation chained equations (MICE) to impute missing values. We will exclude from analyses variables with more than 40% missingness. MICE will be applied for each site separately using the R package mice.

Sample size and power calculation (*Aim 1. Evaluate the effectiveness of a multi-component SDM intervention in a stepped-wedge, cluster-randomized controlled trial on improvement in disease activity, RA knowledge, and adherence.).* Three sites will participate in this study, in which we expect to recruit an average of 11 clinicians per site (33 total). Based on a review of the number of patients with RA diagnoses with an RA clinic visit in year 2019 and an estimated proportion of 40% having active disease, we expect to recruit 4 patients per clinician per site per phase (132 patient surveys). Given our expected sample size (4 visits per clinician per phase, 792 visits total over the six phases) and study design, we will have 83.0% power to detect an average 6-point decrease in RA disease activity from usual care to intervention phases when the standard deviation (SD) is 15 and 91.2% power to detect a 6-point decrease when SD = 13. According to Curtis et al. [38], the minimal clinically important difference for a person with a moderate CDAI is 6 and reported SD≈13. These assumed α = 0.05 in a stepped-wedge cluster randomized trial. All power analyses (Aims 1 and 2) used an ICC = 0.05 [18] and were conducted in PASS 15.0.10 (NCSS, LLC; Kaysville, UT; 2017). For the secondary outcomes of Aim 1, we plan to recruit 6 patients from 11 clinicians at each of the 3 sites, giving a sample size of 198. Since outcomes are binary and participants will be tracked at 3 time points, McNemar’s Test for correlated proportions was used to estimate power comparing two time points after calculating the reduced effective sample size using the ICC. For RA knowledge we assumed 50% of participants will have adequate knowledge at baseline [17], and have 81.1% power to detect an increase to 59% (with 13% discordant pairs) having adequate knowledge at follow-up during the intervention period. For adherence, we assumed 50% adherence at baseline, and have similar power as for RA knowledge. Aim 1 secondary outcome power analyses used α/2 = 0.025 for testing both 6-month and 12-month comparisons with baseline.

Data analysis for Aim 2. Primary analysis of Aim 2 will analyze whether the likelihood of SDM uptake, as measured by CollaboRATE, increased after the intervention launch, using a similar GLMM model as in Aim 1 for CDAI, except with a binary outcome. Decisional conflict using the SURE measure will be analyzed using the same model type as for CollaboRATE. The secondary outcome, OPTION-5, will be evaluated on a random sample of 5 patients per clinician during each of two study periods: pre-intervention and intervention; given the need for audio-recording of clinic visits. We will analyze OPTION-5 using a GLMM with a linear outcome, pre vs. during intervention and site as fixed effects, clinician as a random effect, and other covariates as needed.

Sample size and power calculation (Aim 2). For the primary outcome, we calculated the power to detect differences in varying proportions of top CollaboRATE scores for control and intervention patient encounters in a SWCRT. We will have 85% power to detect a change from 50 to 64% of top CollaboRATE scores with 792 patient encounters. Previous studies have shown widely varying top CollaboRATE scores [41, 43, 48]. The same power analysis holds for decisional conflict using the SURE measure. We plan to use OPTION-5 on 5 patient encounters per clinician in both the control and intervention periods (330 encounters total). Reported OPTION-5 SD’s range from 9.7 to 15.9 [16, 45, 49]. With SD = 12, we have 87.7% power to detect a 5-point mean difference in the control and intervention periods. With only 264 encounters, we still have 82.3% power to detect the same difference. All Aim 2 power analyses used *α* = 0.05.

Data Analysis Aim 3*:* As they are completed, transcribed interviews will be reviewed and discussed by the research team to develop a coding schema for use in analysis. This process of iterative reflection on the data will begin as soon as pre-implementation interviews are completed at one site and will continue as sites are added and as implementation progresses. The coding schema will include both deductive domains and constructs from the CFIR model and additional unanticipated inductive/abductive concepts developed through regular research team discussion. The coding schema will be applied to transcript text by a coding team that will extract data to a matrixed template. All transcripts will be coded by one primary coder and reviewed by a secondary coder, with differences in interpretation discussed and resolved in regular team meetings. This method iteratively builds consensus on patterns in the data while also ensuring multiple interpretations are considered. Analyses will pay attention to the timing and site location of data collected; some findings may focus on issues identified prior to implementation and using only pre-intervention data, while other analyses will draw on data from the entire study period. Analyses will emphasize identifying potential practices impacting the success of implementation of the intervention during this trial, as well as factors likely to influence the success of adoption in future sites.

### Data monitoring

This study includes a data monitoring workgroup (DMW) composed of the principal investigator, study coordinator, and a co-investigator at the main site. The DMW meets monthly to review data management at all sites. Reference to it is in the study protocol; given minimal risk and IRB exempt status, this information is available upon request.

### Veteran engagement group

The PI (Barton) has extensive experience incorporating the patient experience into the development of RA Choice, and in the conduct of RA patient focus groups on treatment discussions [36] and goals [50].

For this project, the PI’s local Veteran Engagement Group (VEG) provided feedback on the proposal and, more specifically, on the patient engagement components of the intervention and how the materials should appear in clinic during the intervention. In addition to this feedback, we will involve veterans/patient input in the following ways:

- Regular presentations to the central site VEG to review potential barriers and challenges to recruitment, study procedures, and interpretation of results at the end of recruitment.
- Inform grant proposal with data from prior RA focus groups (including one on goals with veterans)
- Include measures of patient experience in Aim 2

## Discussion

This study is the first to test and implement a multicomponent intervention to facilitate SDM for persons with RA across geographically diverse sites. Results from this study can be used to inform future larger-scale implementation of interventions to foster SDM for persons with RA in VA- and non-VA settings. These results can also inform implementation of SDM not only for changing or escalating therapy but also to promote discussions around tapering biologic or targeted synthetic DMARDs [51, 52] as well as non-pharmacologic therapies for RA [53]which call for SDM.

In the instance of achievement of improvement in disease activity and patient-reported experience of SDM after completion of this trial, future studies could assess different implementation strategies, cost-effectiveness of the interventions, and sustainability (is SDM maintained or integrated across clinics, how is SDM maintained with change in clinicians, e.g., rotating fellows in training). These questions could be explored in future studies.

### Dissemination

Results from this study will be presented at national and international meetings in rheumatology, health services, and health communication, and published in peer-reviewed journals. Intervention materials and clinician trainings will be shared with VA rheumatologists both locally and nationally and be made available electronically for download and free use. We will share study results with our local and national Veteran Engagement Groups.

### Trial status

Participant enrollment in the study began in September 2022, and we estimate enrollment will be completed by October 2025. The current study protocol is version 9 from July 2024.

## Supplementary Information

Additional file 1. RA Medication Summary.

Additional file 2. RA Choice decision aid.

Additional file 3. RA knowledge questionnaire.

## Data Availability

Not applicable.
