# Supplementary material for: Implementation of shared decision making in rheumatoid arthritis: study protocol for RAiSeD (Rheumatoid Arthritis Shared Decision Making) stepped wedge, cluster-randomized trial
Source: Trials. 2025 Sep 29;26:381. doi: 10.1186/s13063-025-09015-1 (PMC12482761; doi:10.1186/s13063-025-09015-1)
Supplement: Supplementary file 2 — Additional file 2. RA Choice decision aid. [file 13063_2025_9015_MOESM2_ESM.pdf]

Your Guide to

Rheumatoid Arthritis Medicines

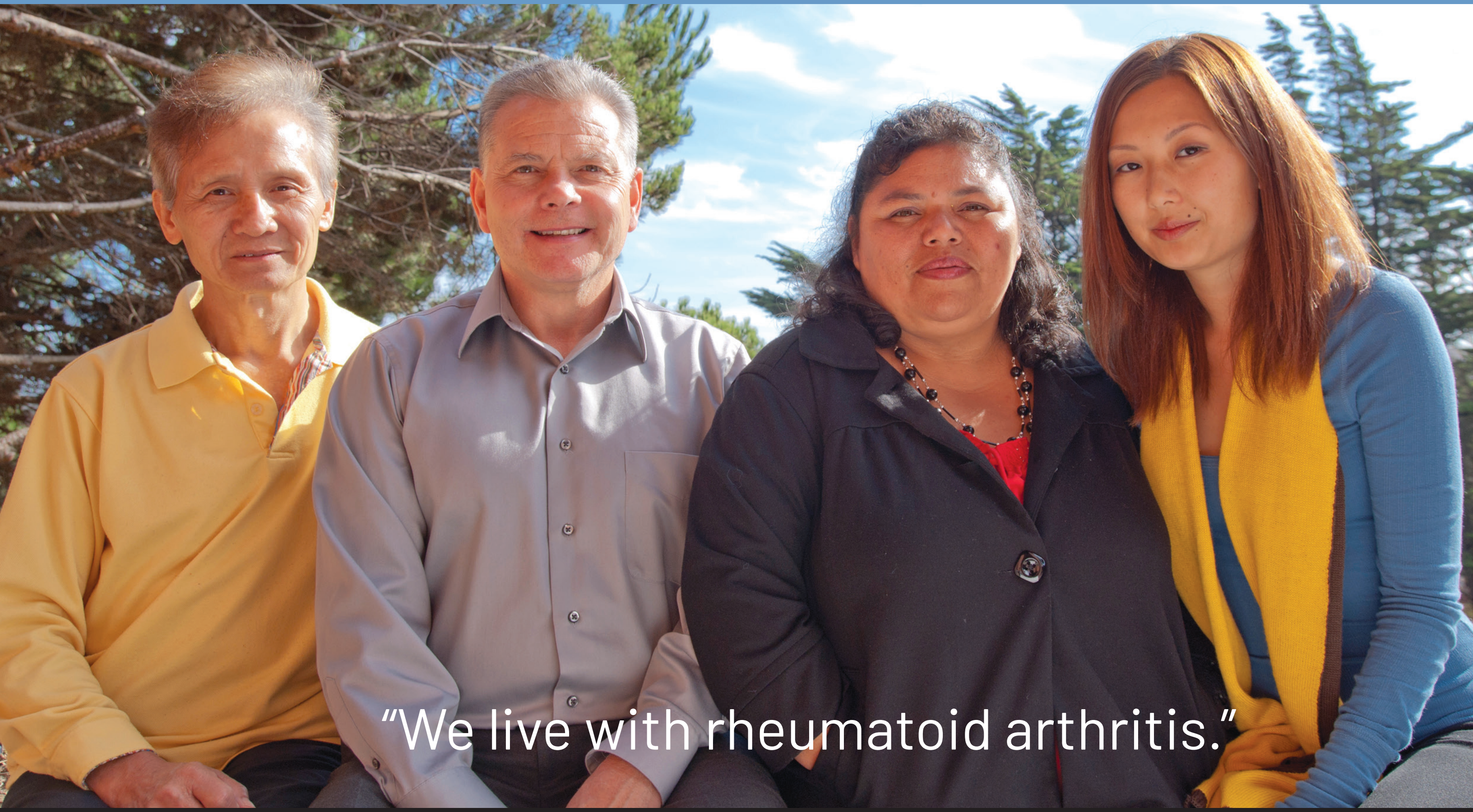

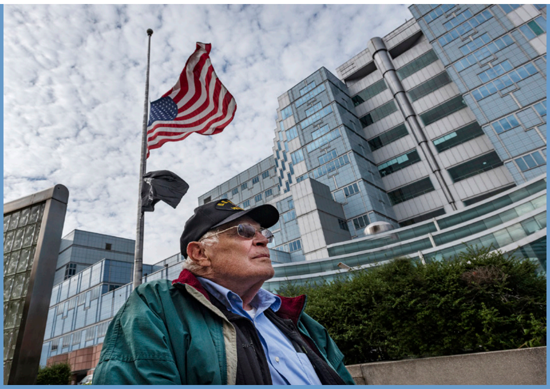

VA | U.S. Department of Veterans Affairs

Veterans Health Administration

VA Portland Health Care System

Your Guide to

Rheumatoid Arthritis Medicines

Chapter 1 - Page 4

What is rheumatoid arthritis (RA)?

Chapter 2 - Page 6

What can RA medicines do for you?

Chapter 3 - Page 10

How can you get the most out of your doctor's visit?

Chapter 4 - Page 12

How can being active and eating well help your RA?

Chapter 5 - Page 14

Additional information about RA medicines

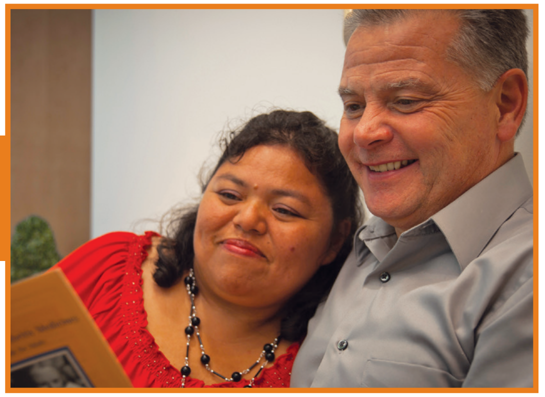

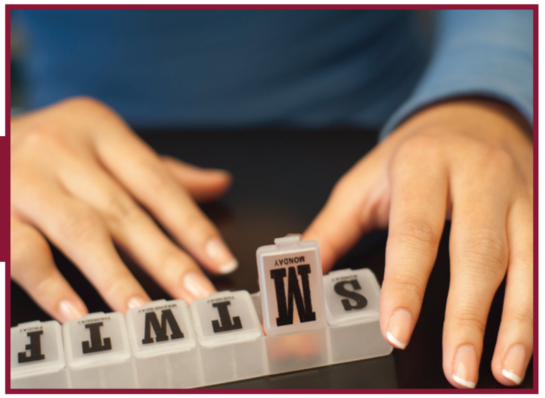

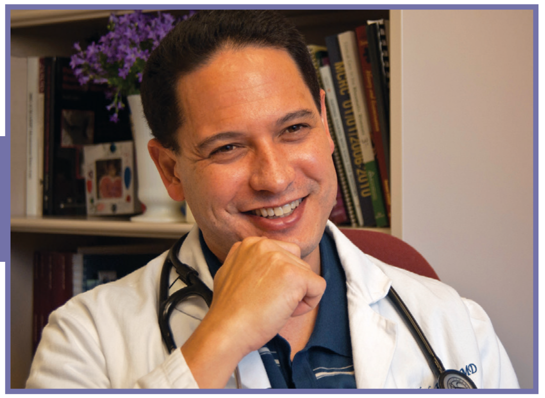

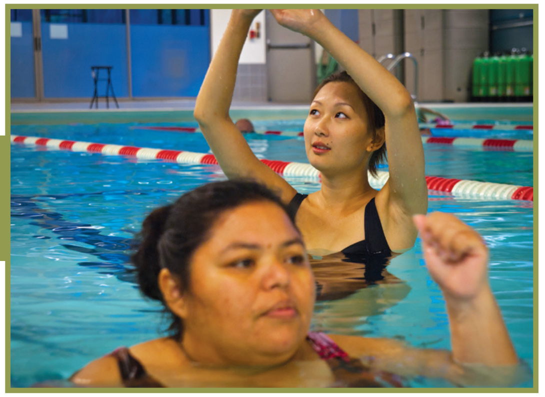

This guide will help you:

- Learn about rheumatoid arthritis (RA).
- Understand what RA medicines can do for you.
- Choose the right medicine for you with help from your doctor.
- Have a successful doctor's visit.
- Be active and eat well with RA.
- Find more information about RA.

"The doctor told me that the arthritis, this disease, 'you're going to conquer it, it's not going to conquer you.'" - Susan, age 50

Chapter 1

What is rheumatoid arthritis (RA)?

"I even flew back to China to try to get a cure for it because... I thought there was a cure." - Joan, age 25

What is RA?

When you have rheumatoid arthritis, your immune system, which usually fights infection, attacks the lining of your joints and causes inflammation. Inflamed joints can be swollen, stiff, and painful. The small joints of your hands and feet are usually affected first.

Once you have RA, it never goes away. You have it for life. Medicines can help with pain and stiffness in your joints and prevent permanent damage to your joints.

How do you get RA?

No one knows exactly what causes RA. It is most likely a combination of your genes and the environment. Smoking also increases your chances of getting RA.

What can RA do to your body?

- Cause swelling, warmth and pain in your joints
- Permanently damage your joints
- Cause inflammation of your eyes, blood vessels, or lungs
- Weaken bones (osteoporosis)
- Cause anemia (low blood cell counts)
- Make you feel very tired
- Give you a fever
- Cause changes in your weight
- Make you feel sad or depressed

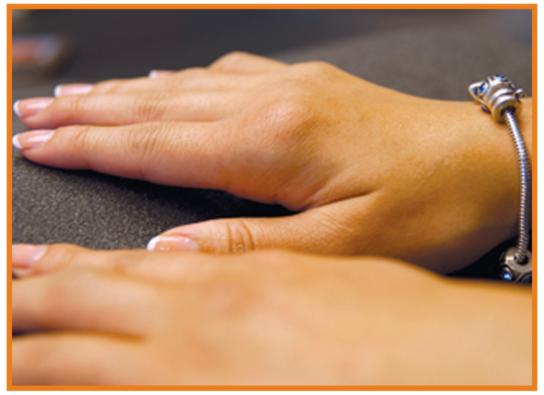

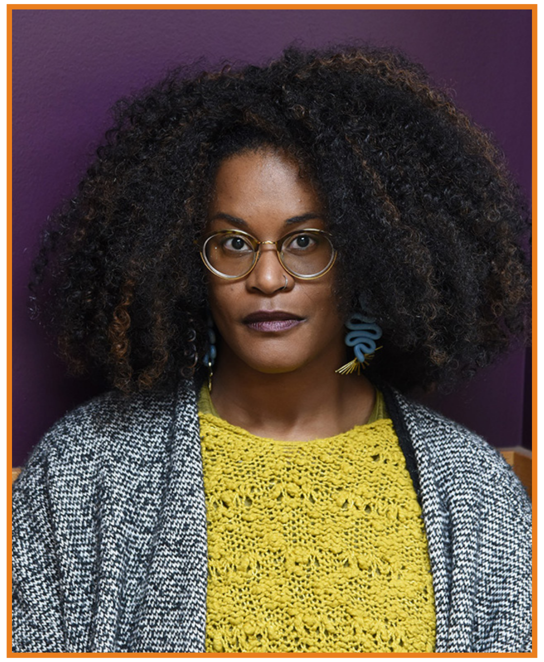

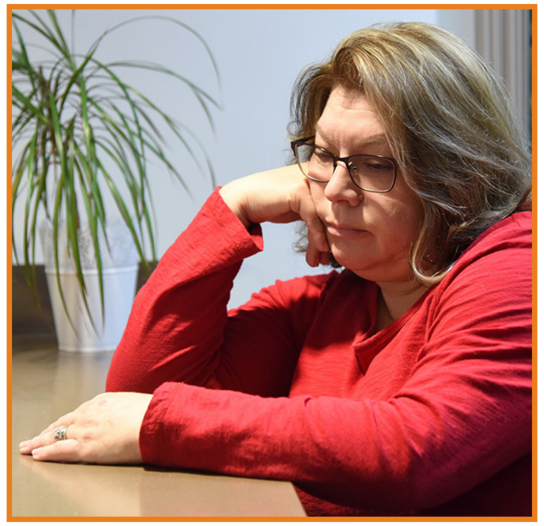

Who gets RA?

- Anyone can get RA.
- You can get it at any age.
- Women are 2-3 times more likely than men to get RA.

Is there a cure for RA?

No medicine can cure RA, but medicine can slow down damage to joints and ease pain. Most people have to take pills, shots, or medicine by vein (IV or intravenously).

When should you begin to take medicine for RA?

- It is important to take medicine for RA as soon as a doctor tells you that you have it.
- People who take medicine early are able to do everyday activities better and are less likely to have permanent damage to their joints than those who wait.

Do people with RA have other health problems?

Yes, some do. You may have a greater risk of:

- Heart disease
- Certain types of cancer (lymphoma – a blood cancer)
- Broken bones from osteoporosis

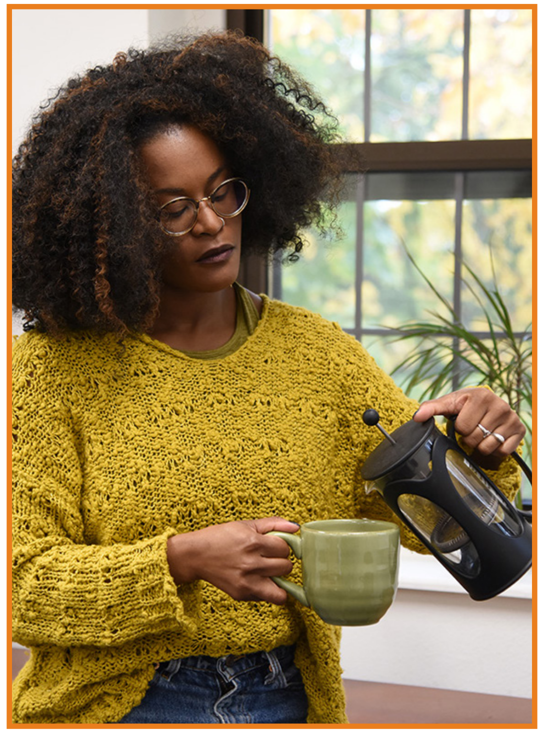

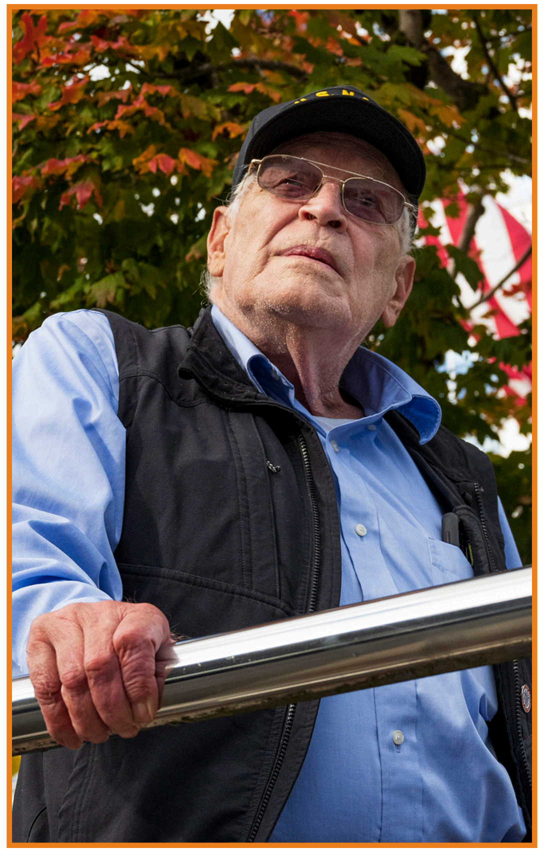

Chapter 2

What can RA medicines do for you?

There are many different kinds of medicine for RA. This guide talks about the medicines that can reduce swelling and help prevent damage to the joints. It does not talk about over-the-counter medicines (like Tylenol® or Advil®), pain medicines, or steroids, which help treat the symptoms of RA but do not prevent damage.

RA medicines can:

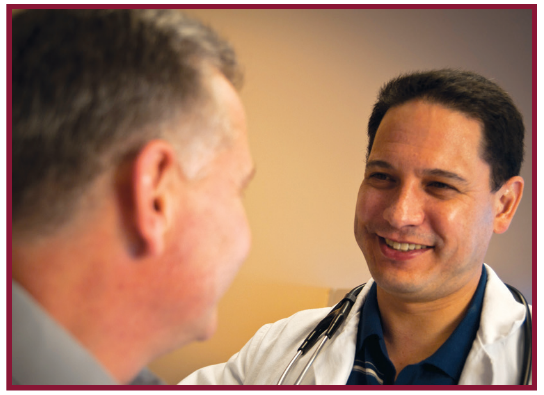

- Help your pain and swelling in your joints
- Slow down changes to your joints, most often in your hands and feet
- Improve the quality of your life

Your doctor can help you pick the best medicine or medicines for you. If one medicine does not work, you have other choices.

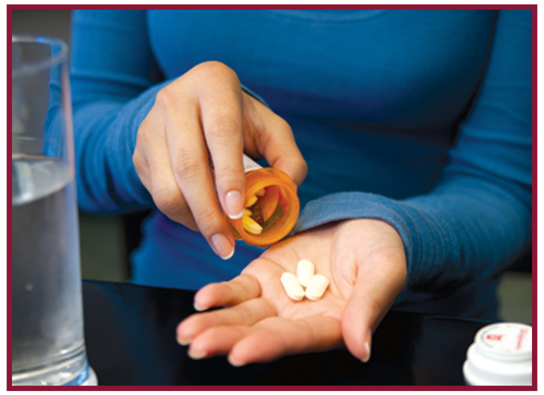

"Sometimes I do not understand why I have to take medicine, but then I am reminded of what I go through without it." - Maria, age 40

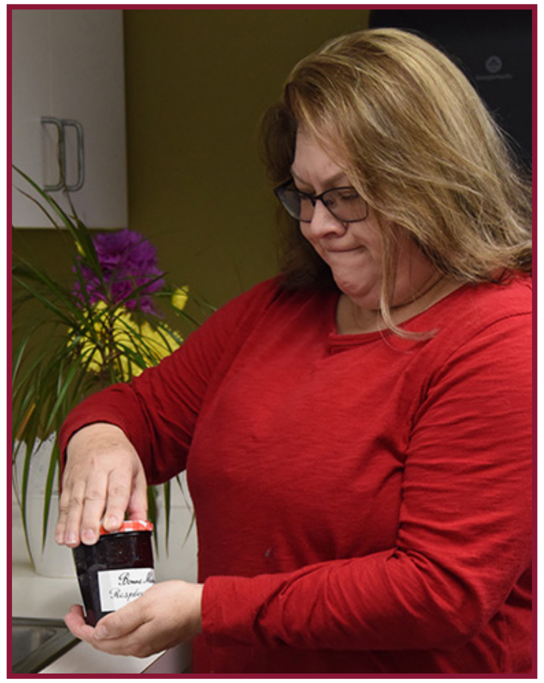

What can happen to the hands and feet of people with RA?

Normal joints	Mild to moderate swelling in joints	Permanent damage to joints
More likely if on medicine early	More likely if medicine not working	More likely without medicine
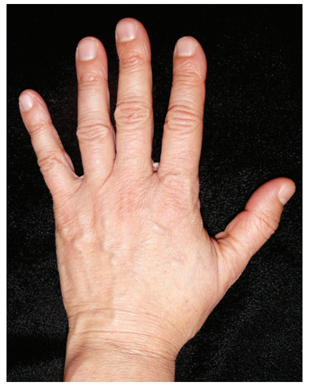	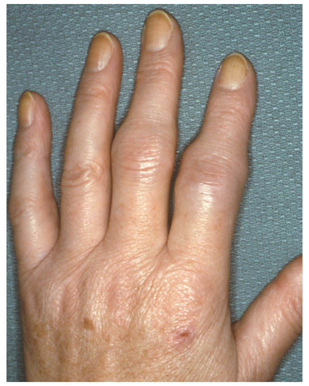	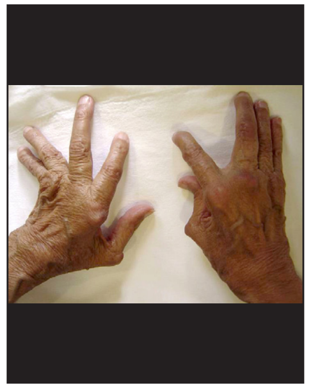
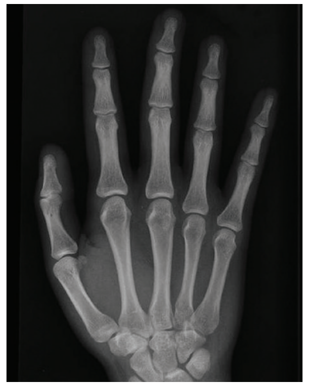	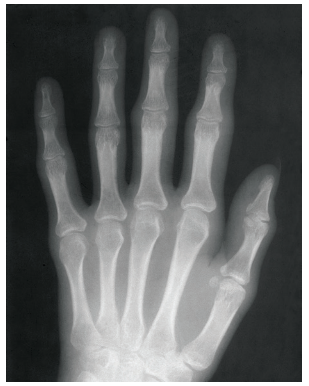	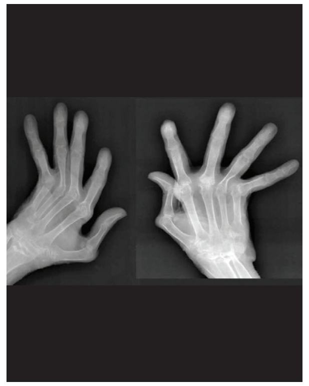
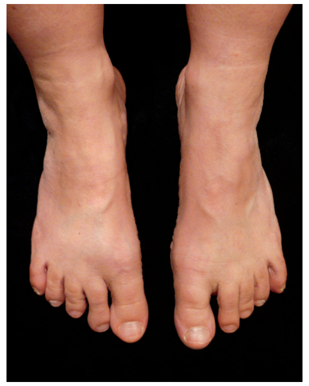	Permanent damage is less likely if the disease is controlled with medicine. This is why it is important to take medicine for RA as soon as your doctor tells you that you have it.	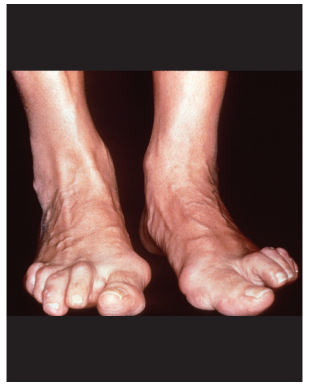
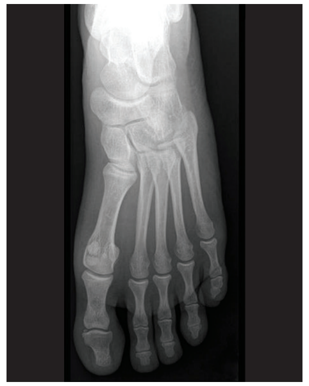	**Photographs reprinted with permission from the American College of Rheumatology**	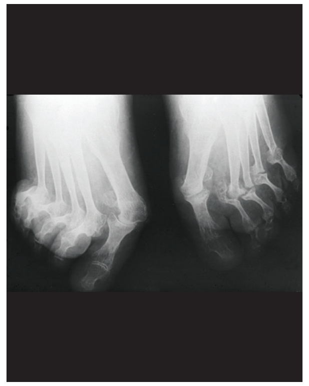

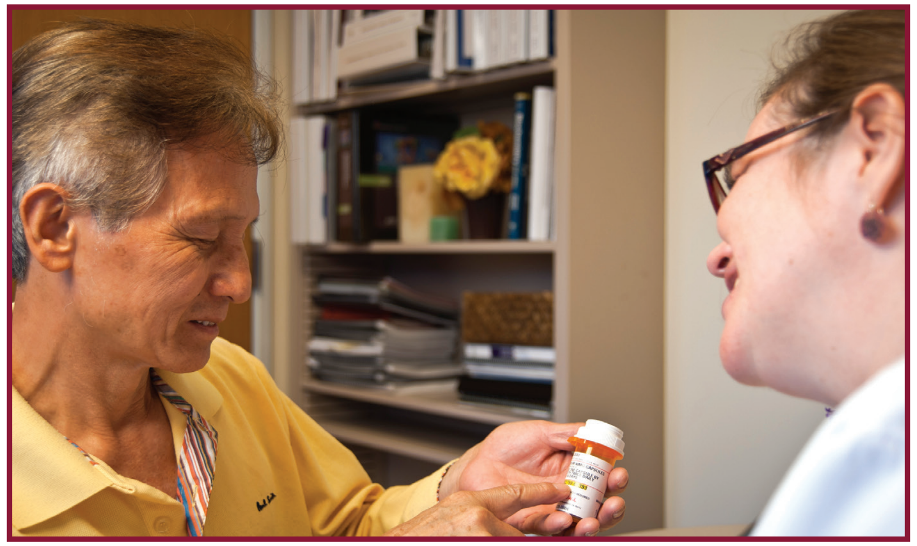

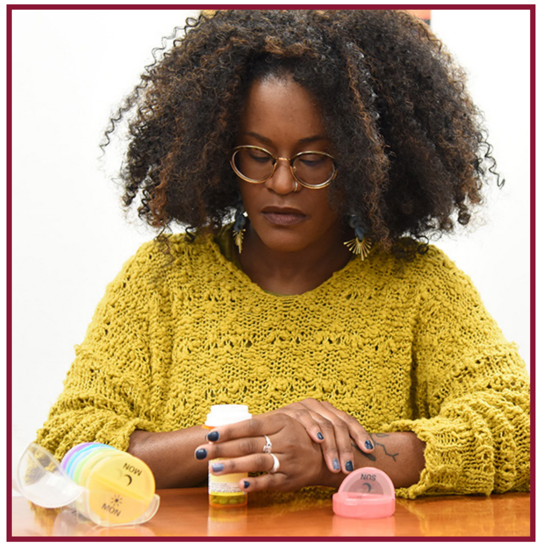

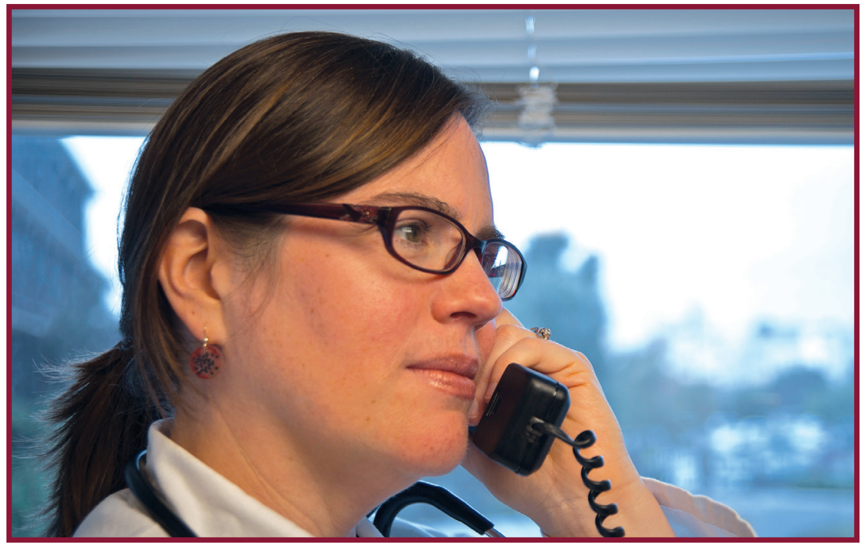

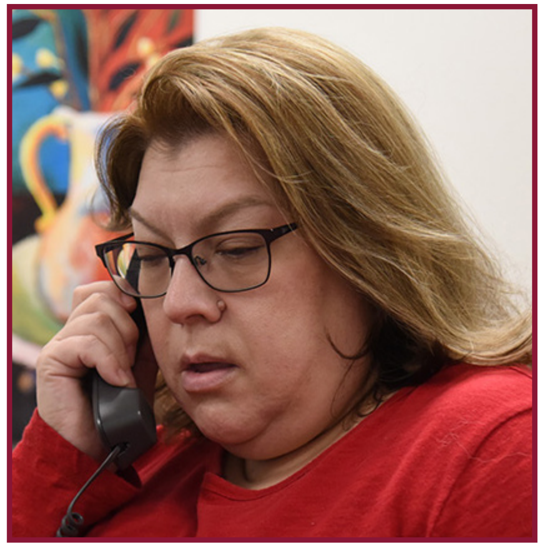

Finding the right medicine for you:

- There are many different medicines for RA.
- You can take RA medicines by:

Mouth (orally)	Taken at home
Shot (in the belly or thigh)	Taken at home | given at doctor's office or hospital
Vein (IV - intravenously)	Given at doctor's office or hospital

Medical research shows that most RA medicines all work about the same on their own. Most people with RA need to take more than one medicine. Taking two or more medicines together (such as pills and a shot) often works better than just taking one alone. Doctors often combine the RA medicines talked about in this guide with steroids and anti-inflammatory pills.

You may have to try different RA medicines to find the right one for you. Every patient is different. You and your doctor will not know if a medicine will work for you until you try it.

"The medicine really can help, and there are many you can choose from."
- José, age 69

What are some of the side effects from RA medicines?

Like most other medicines, RA medicines also come with side effects. Most people will have at least one side effect from an RA medicine.

Each medicine has different side effects. Your doctor should talk with you about the side effects of each medicine. If you do not understand the side effects, talk to your doctor. Some common side effects from RA medicines include stomach upset (such as nausea or diarrhea), cold symptoms, or rash.

Some very rare serious side effects include:

- Infections that require you to be in the hospital and get antibiotics by vein
- Some kinds of cancer (skin or blood)

Visits with your RA doctor and blood tests can help identify side effects from the medicines. It is important to work together with your RA doctor to help prevent side effects.

Having a side effect does not mean you have to stop the medicine. You should always ask your doctor if you have questions and before you stop a medicine. Most side effects are not emergencies, but call your doctor or nurse immediately if you have a fever or new cough.

Are you thinking about having a baby?
Some RA medicines can cause serious birth defects. You should talk to your doctor before you or your partner plan a pregnancy.

How can you get the most out of your doctor's visit?

Getting ready for your doctor's visit.

- Bring all of your medicines to your doctor's visit. Include vitamins, over-the-counter, and herbal med icines.

At your visit, it is important to tell your doctor about:

- Your list of questions
- Any pain
- Any stiffness (tightness in your joints)
- How well you sleep
- Your mood (how you feel)
- Your ability to do daily activities, like get dressed, cook, or bathe
- Problems getting or taking medicine
- Side effects from medicine
- Visits to the hospital, emergency room, or other doctors

Sharing this information will help you and your doctor make good decisions together.

Tell your doctor if you are pregnant or if you or your partner are planning a pregnancy.

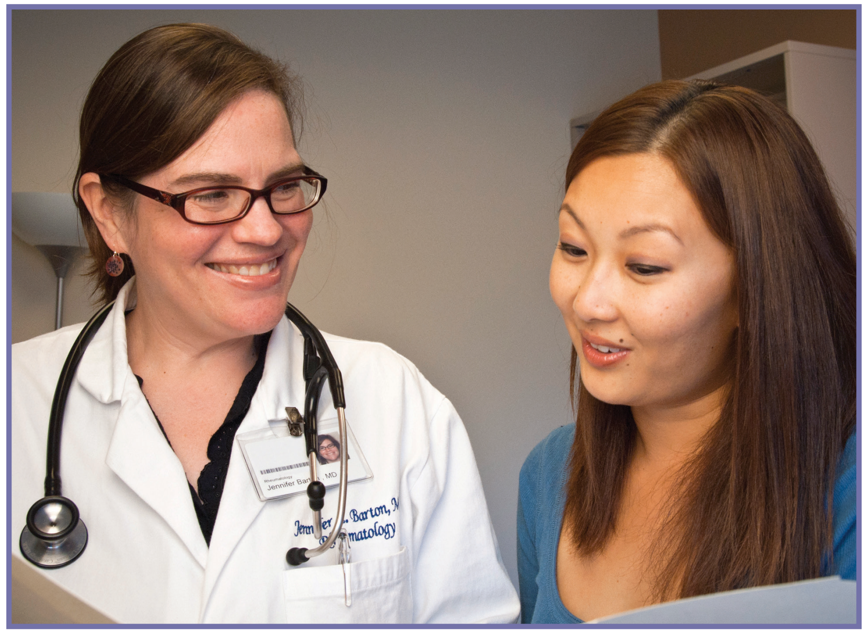

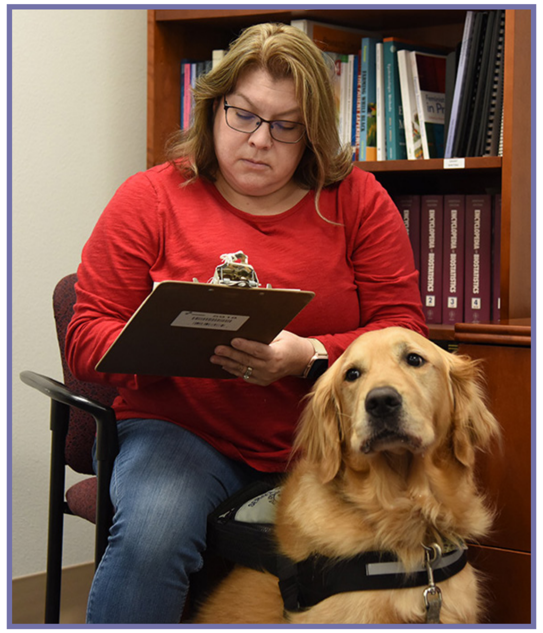

"I feel really good because the doctor and I have a lot of dialogue. And the doctor always has answers to my questions. I have confidence in my doctor." - Eva, age 32

If you smoke...

Smoking can make rheumatoid arthritis worse and make the medicines less effective. Quitting is not easy, but it's not impossible. Many people have stopped smoking, and you can too.

When you are ready to quit:

- Ask your health care clinician or pharmacist about medicines to help.
- Pick a "quit date".
- Tell your friends and family you will stop smoking on your quit date.
- On your quit date, throw away your cigarettes, ashtrays, lighters, matches, and anything else that makes you think about smoking. The harder it is to get a cigarette, the easier it will be to quit.

"When I quit smoking, I feel better." - Charlie, age 37

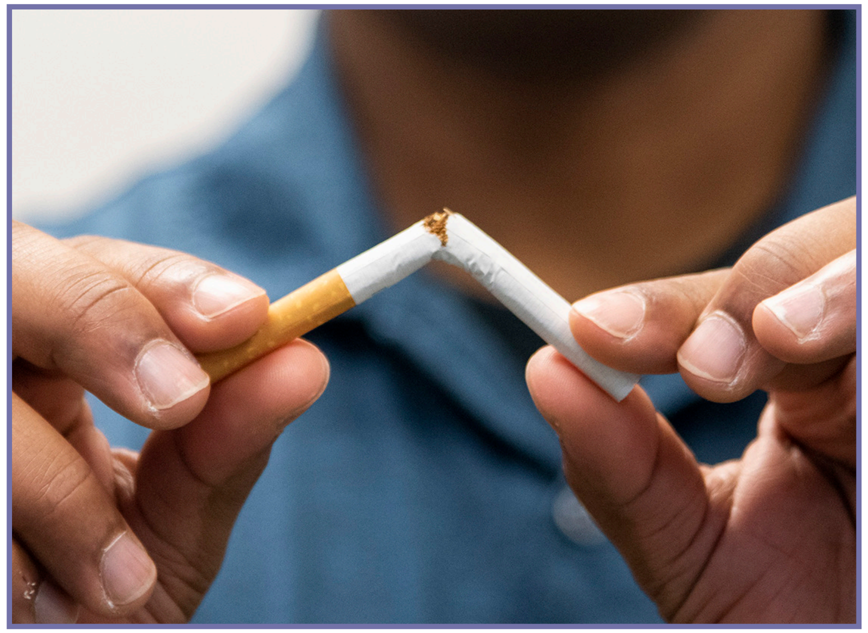

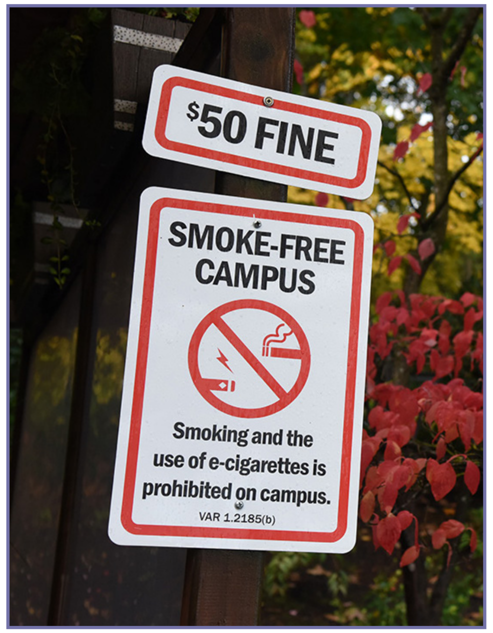

Chapter 4

How can being active and eating well help your RA?

Being physically active may help your RA symptoms by reducing pain and improving physical function.

There are many different kinds of exercise that you can do to help your RA:

- Aerobic exercise, such as walking or dancing
- Strength training, such as lifting light weights
- Water exercise, such as swimming or special arthritis water exercise classes

In addition to doing exercise on your own, your doctor may send you to an occupational therapist or a physical therapist.

- Occupational therapists can give you advice on how to do everyday activities – such as dressing, cooking, and cleaning – with less pain.
- Physical therapists can help with joint function, strength and overall health. Therapists may use exercise, heat/cold, or massage to decrease pain and improve your ability to do everyday activities.

"You can look to other ways to making yourself feel better. I make myself exercise every day. Just being out in the morning helps me." - Ed, age 49

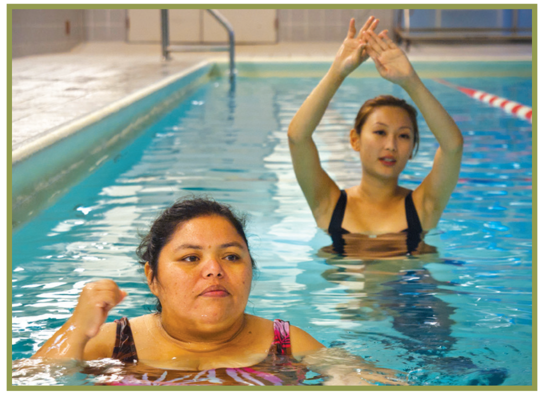

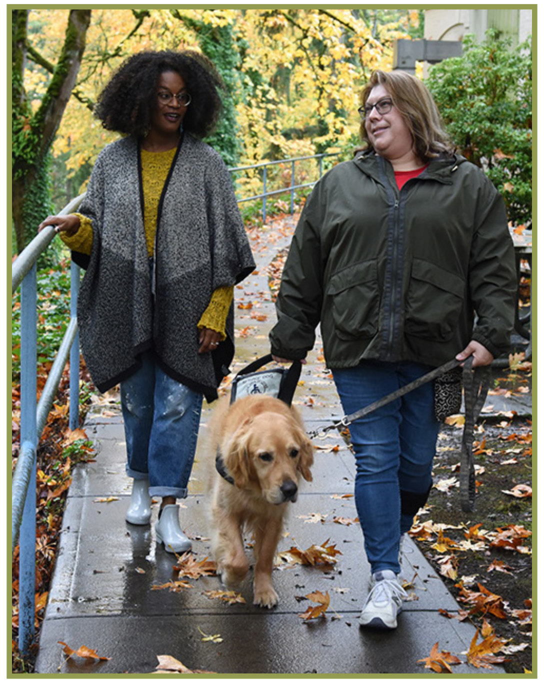

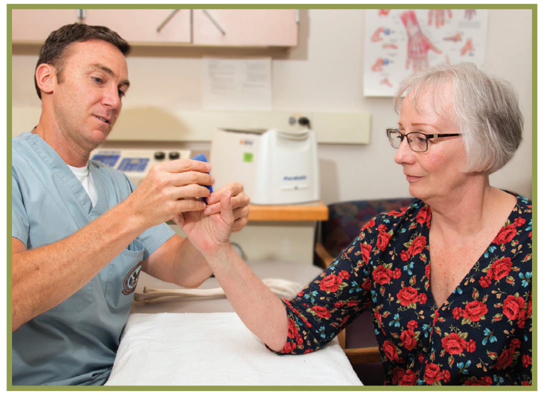

How can eating right help me?

People with RA are at higher risk for heart disease. Eating a healthy diet is one of the most important things to help you have a healthy heart and live longer.

What are some ways to eat healthy?

- Eat smaller portions
- Prepare food in healthy ways
- Eat more fruits and vegetables
- Get calcium and vitamin D from milk, yogurt, and leafy green vegetables

You do not have to be hungry or stop eating your favorite foods.

How can you benefit from healthy eating?

Healthy eating can:

- Give you more energy
- Help you lose weight
- Lower your blood pressure and cholesterol
- Lower your risk of having a heart attack
- Keep your bones healthy and strong

The healthy plate: Think of your plate in different sections. One half is for vegetables and fruits and the other half for proteins (such as meat, fish, eggs, or beans) and grains (such as pastas, breads, tortillas, or rice).

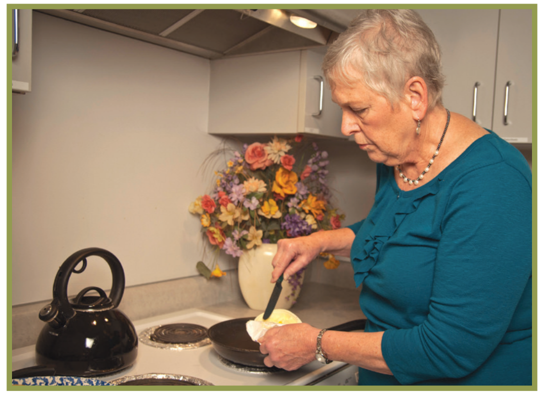

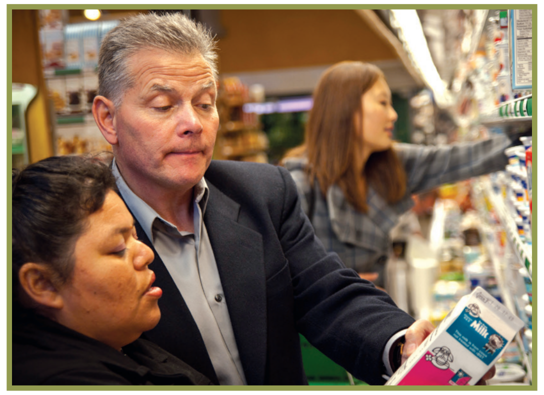

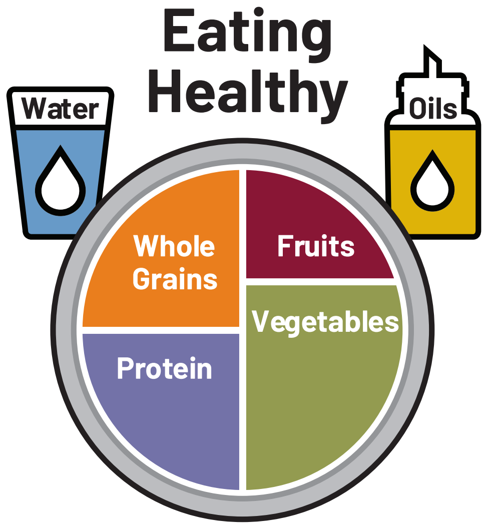

Additional information about RA medicines

Many of these medicines should NOT be taken if you are considering pregnancy, have active tuberculosis (TB) or exposure to TB, drink alcohol, or have liver disease. If you are pregnant or considering pregnancy in the next year, it is very important to talk to your doctor about these issues before you choose a medicine.

Class	Medication	Method	Don't Take If	How Often
csDMARD	**Methotrexate**	Pill, Injection	Pregnancy, Alcohol, Liver Disease, Breastfeeding	Once weekly
csDMARD	**Leflunomide** (Arava)	Pill	Pregnancy, Alcohol, Liver Disease, Breastfeeding	Once daily
csDMARD	**Hydroxychloroquine** (Plaquenil)	Pill		Once daily
csDMARD	**Sulfasalazine** (Azulfidine)	Pill		Twice daily
Anti-TNF	**Adalimumab*** (Humira)	Injection	Tuberculosis	Every 2 weeks
Anti-TNF	**Certolizumab*** (Cimzia)	Injection	Tuberculosis	Every 2 weeks
Anti-TNF	**Etanercept*** (Enbrel)	Injection	Tuberculosis	Once weekly
Anti-TNF	**Golimumab*** (Simponi)	Injection, Infusion	Tuberculosis	Every 4 weeks (Injection)<br>Every 8 weeks (Infusion)
Anti-TNF	**Infliximab** (Remicade)	Infusion	Tuberculosis	Every 8 weeks
CTLA 4Ig Cell-blocker	**Abatacept*** (Orencia)	Injection, Infusion	Tuberculosis, Pregnancy	Once weekly (Injection)<br>Every 4 weeks (Infusion)
Anti-IL-6	**Sarilumab*** (Kevzara)	Injection	Tuberculosis, Pregnancy, Liver Disease	Every 2 weeks
Anti-IL-6	**Tocilizumab*** (Actemra)	Injection, Infusion	Tuberculosis, Pregnancy, Liver Disease	Every other week (Injection)<br>Every 4 weeks (Infusion)
Anti-JAK	**Baricitinib** (Olumiant)	Pill	Tuberculosis, Pregnancy, Liver Disease, Breastfeeding	Once daily
Anti-JAK	**Tofacitinib** (Xeljanz)	Pill	Tuberculosis, Pregnancy, Liver Disease, Breastfeeding	Twice daily
Anti-JAK	**Upadacitinib** (Rinvoq)	Pill	Tuberculosis, Pregnancy, Liver Disease, Breastfeeding	Once daily
Anti-CD20	**Rituximab** (Rituxan)	Infusion	Tuberculosis, Pregnancy**	Every 4-6 months++

Symbol Key
- Tuberculosis
- Pregnancy
- Alcohol
- Liver Disease
- Breastfeeding

***Refrigerate** **Please tell your doctor if you are planning pregnancy. ++First dose followed by second dose 2 weeks later through every 4-6 months.

My important contacts and phone numbers

Clinician(s): ______________________________

Phone Number: ______________________________

Pharmacy: ______________________________

Phone Number: ______________________________

Nurse or Facilitator: ______________________________

Phone Number: ______________________________

Supports (family member, friend, care taker, etc.): ______________________________

Phone Number: ______________________________

Write down all the questions you have for your doctor before the visit:

____

____

____

____

Make a list of your side effects from your medicines:

____

____

____

____

VA | U.S. Department of Veterans Affairs
Veterans Health Administration
VA Portland Health Care System

Where can you get more information about RA?

Ask your doctor if you have questions.

For more information on RA medication and side effects:

National Institute of Arthritis and Musculoskeletal and Skin Diseases (NIAMS)
www.niams.nih.gov

- Fact sheets available in English, Chinese, Spanish
- Audio publications also in English, Chinese, Spanish

American College of Rheumatology website (under "Patient Resources"):
www.rheumatology.org

- Information available in Spanish and English

Arthritis Foundation:
www.arthritis.org

- Information available in Spanish: http://www.arthritis.org/español

PALS - Patient Activated Learning System
www.palsforhealth.com
Developed at Weill Cornell Medicine by Monika Safford, MD, an internist, and Jeff Curtis, MD, a rheumatologist. Dr. Safford is the Chief of General Internal Medicine at Weill Cornell Medicine and Dr. Curtis is a Professor of Medicine at the University of Alabama at Birmingham.

Where did the information in this guide come from?

This guide was adapted from Rheumatoid Arthritis Medicines: A Guide for Adults, produced in 2008 by the Eisenberg Center at Oregon Health & Science University with funding from the U.S. Agency for Healthcare Research and Quality (AHRQ). The current version is an update of the "Your Guide to Rheumatoid Arthritis Medicines" originally developed by Jennifer Barton MD, Edward Yelin PhD, Gina Evans-Young, Laura Trupin MPH, John Imboden MD, and Dean Schillinger MD, with input from Dana Ragouzeos and Jennie Anderson MS, and funding from AHRQ. People with RA and rheumatologists helped to write and review this guide. Funding for the updated guide is from the Louis Gerlinger, Jr., and Beatrice Lee Gerlinger Trust Research Award (Principal Investigator: Dr. Barton).

Photography by Lena Zhang, Andrew Lin, and D. Michael Moody. Design by Timothy Morse.
