# Supplementary material for: Implementation of shared decision making in rheumatoid arthritis: study protocol for RAiSeD (Rheumatoid Arthritis Shared Decision Making) stepped wedge, cluster-randomized trial
Source: Trials. 2025 Sep 29;26:381. doi: 10.1186/s13063-025-09015-1 (PMC12482761; doi:10.1186/s13063-025-09015-1)
Supplement: Supplementary file 3 — Additional file 3. RA knowledge questionnaire. [file 13063_2025_9015_MOESM3_ESM.pdf]

Appendix 6. Rheumatoid arthritis medication knowledge survey

1. **Once you have rheumatoid arthritis, how long does it last?**

   – a. For 6 months

   – b. For 3 weeks

   – c. For a few days

   – d. For life

**Mark the following sentences as true (T) or false (F):**

2. **Medicines can cure rheumatoid arthritis.**
3. **Medicines are the main treatment for rheumatoid arthritis.**
4. **Rheumatoid arthritis can affect the eyes.**
5. **RA medicines can be taken as a pill, a shot or in the vein.**
6. **RA medicines can never be combined.**
7. **RA medicines decrease swelling in joints and relieve pain.**
8. **Most people only need RA medicines for a couple of days.**
